# DNA/Magnetic Nanoparticles Composite to Attenuate Glass Surface Nanotopography for Enhanced Mesenchymal Stem Cell Differentiation

**DOI:** 10.3390/polym14020344

**Published:** 2022-01-17

**Authors:** Ilnur Ishmukhametov, Svetlana Batasheva, Elvira Rozhina, Farida Akhatova, Rimma Mingaleeva, Artem Rozhin, Rawil Fakhrullin

**Affiliations:** Institute of Fundamental Medicine and Biology, Kazan Federal University, Kreml uramı 18, 420008 Kazan, Republic of Tatarstan, Russian Federation; irishmukhametov@gmail.com (I.I.); svbatasheva@gmail.com (S.B.); akhatovaf@gmail.com (F.A.); RNMingaleeva@kpfu.ru (R.M.); rozhinartemkzn@gmail.com (A.R.)

**Keywords:** nanotopography, iron oxide nanoparticles, DNA, hTERT-transduced mesenchymal stem cells, osteogenesis, adipogenesis, chondrogenesis

## Abstract

Mesenchymal stem cells (MSCs) have extensive pluripotent potential to differentiate into various cell types, and thus they are an important tool for regenerative medicine and biomedical research. In this work, the differentiation of hTERT-transduced adipose-derived MSCs (hMSCs) into chondrocytes, adipocytes and osteoblasts on substrates with nanotopography generated by magnetic iron oxide nanoparticles (MNPs) and DNA was investigated. Citrate-stabilized MNPs were synthesized by the chemical co-precipitation method and sized around 10 nm according to microscopy studies. It was shown that MNPs@DNA coatings induced chondrogenesis and osteogenesis in hTERT-transduced MSCs. The cells had normal morphology and distribution of actin filaments. An increase in the concentration of magnetic nanoparticles resulted in a higher surface roughness and reduced the adhesion of cells to the substrate. A glass substrate modified with magnetic nanoparticles and DNA induced active chondrogenesis of hTERT-transduced MSC in a twice-diluted differentiation-inducing growth medium, suggesting the possible use of nanostructured MNPs@DNA coatings to obtain differentiated cells at a reduced level of growth factors.

## 1. Introduction

Mesenchymal stem cells (MSCs) are adult stromal cells, which have extensive pluripotent potential to differentiate into various specialized cells when cultured in vitro, including chondrocytes, adipocytes and osteoblasts [1,2]. The in vitro differentiated cells are an important tool of biomedical research, such as disease modelling, drug screening and investigations of cellular regulatory mechanisms [3,4]. As a result of in vitro MSCs differentiation, large amounts of differentiated cells can be obtained without raising many ethical issues [5,6], while MSCs self-renewal ability and relatively low immunogenicity [7,8] are advantageous for their application in stem cell therapy [9].

The sources of MSCs are numerous, including adipose and bone tissue, umbilical cord blood, placenta, dental pulp and many other tissues of an adult [10,11]. To induce the cell differentiation in vitro, stem cells are exposed to culture media supplemented with specific growth factors [12,13]. Such media can be rather expensive; therefore, the development of cheaper approaches to promote differentiation of cells is desirable. For instance, the ability of forskolin-modified halloysite nanotubes within porous hydrogel scaffolds to induce the in vitro osteodifferentiation of MSCs without using growth factors was demonstrated recently [14].

It is known that not only chemical signals, but also mechanical signals from the extracellular matrix (ECM) play an important role in cell differentiation in the human organism [15,16], which potentially makes it possible to create substrates that provoke cell differentiation without using or using a lower concentration of morphogenetic growth factors [17]. The ECM has a hierarchical structure of proteoglycans and fibrous proteins: collagens, elastins, fibronectins and laminins [18]. Cell adhesion to the ECM is associated with specific molecules, mainly integrins, discoidin domain receptors and syndecans. The cell membrane interacts with fibrils using adhesive molecules, mainly integrin, a transmembrane receptor that is associated on the inner side with adapter proteins involved in signaling pathways. Thus, various nanotopographic features of the matrix can act as a physical signal for initiating subsequent intracellular events, including changes in adhesion, morphology, and gene expression [19,20,21,22]. It is also known that biophysical properties of substrates, such as stiffness and viscosity, as well as biochemical factors, could alter the behavior of MSCs (i.e., proliferation, differentiation and migration) [23].

To mimic ECM properties, artificial cell scaffolds are fabricated using various fibers or nanoparticles embedded in a polymeric matrix [24,25,26,27,28]. The nanoparticles reinforce the mechanical properties of polymer substrates in addition to imparting them with specific topographic features. The cell behavior is influenced not only by the number and distribution patterns of nanoparticles on the surfaces, but also by their size [29], shape [30], chemical composition [31], as well as other substrate properties [32,33]. For example, silver nanoparticles are often used to modify polymer matrices to impart them with antibacterial properties, given their low toxicity to mammalian cells, which was shown in several cell lines [34,35]. Silver nanoparticles incorporated in polymer brushes were used to modify the glass surface and had no cytotoxic effect towards normal keratinocytes but demonstrated a slight anti-cancer effect on the WM35 cell line derived from the primary melanoma site [36].

Recently, magnetic iron oxide nanoparticles (MNPs) have appeared as a perspective component of tissue engineering scaffolds. In addition to the features that are characteristic of many nanomaterials (i.e., large surface area, colloidal stability and the possibility of surface functionalization) [37], MNPs also show moderate activity in different cell types [38,39,40]. MNPs have low cytotoxicity and are actively used to treat iron deficiency anemia [41]. They were widely used as contrast agents for non-invasive medical visualization in the 1980s−2000s [42,43,44,45]; although the current use of MNPs for magnetic resonance imaging has practically ceased due to low resolution and undesired accumulation in tissues [46]. A stimulating effect of MNPs on the process of cell differentiation was shown [17,47], including a positive effect on osteogenic and chondrogenic transitions of cells [11,26,45,48,49,50]. The synergistic effect of magnetic iron oxide nanoparticles, a magnetic field and a differentiation medium on differentiation of stem cells was found [17].

In nanostructured cell supports, the nanoparticle building blocks are bound together by polymers, which can be synthetic or of natural origin. The proper choice of a polymer is important as some synthetic polyelectrolytes can be rather cytotoxic [51,52], making biopolymers an attractive alternative.

Being a natural polyanion, DNA is a perspective material for various biomedical applications [53] due to its physico-chemical properties, nontoxicity, non- or low immunogenicity [54], the ability to incorporate other compounds [55] and high phosphate content [56]. Due to modern methods of DNA isolation and purification, eukaryotic DNA is now a rather cheap material that can be obtained in high amounts from such sources as fish wastes [57] and vegetable scraps [58]. DNA was proposed as a component for coatings of bone and dental titanium implants [59]. Multilayer DNA coatings are cyto- and histocompatible [56] and may contribute to the deposition of calcium phosphates in the bone environment [60]. In addition, DNA coatings are considered stable [61]. However, the potential of using DNA together with MNPs as components of substrates for in vitro MSCs differentiation into various cell types remains understudied. In this work, the differentiation of hTERT-transduced adipose-derived MSCs (hMSCs) into chondrocytes, adipocytes and osteoblasts on substrates with nanotopography generated by magnetic nanoparticles and DNA was investigated.

## 2. Materials and Methods

### 2.1. Materials

Iron(II) chloride tetrahydrate, iron(III) chloride, 0.25% trypsin-EDTA, 4′,6-diamidino-2-phenylindole (DAPI), phalloidin–tetramethylrhodamine B isothiocyanate (phalloidin-TRITC), 3-isobutyl-1-methylxanthine, dexamethasone, indomethacin, Oil Red O solution 0.5% in isopropanol, 2-phospho-L-ascorbic acid trisodium salt, β-glycerophosphate disodium salt hydrate (99%) and silver nitrate (99%) were obtained from Sigma-Aldrich (Darmstadt, Germany). Citric acid monohydrate was purchased from Acros Organics (Geel, Belgium). Plasmid pWPT-GFP was obtained from Addgene (cat #12255; Cambridge, MA, USA). Dulbecco’s modified eagle medium (DMEM), L-glutamine, and 1% penicillin−streptomycin, dimethyl sulfoxide (DMSO), insulin (human recombinant), Dulbecco’s phosphate-buffered saline (PBS) and ethylenediaminetetraacetic acid disodium salt dihydrate (EDTA; 98.5%) were obtained from PanEco Ltd. (Moscow, Russia). Sodium hydroxide (99.3%) and safranin O were purchased from Dia-M (Moscow, Russia). Heat-inactivated fetal bovine serum (FBS) and StemPro™ Chondrogenesis Differentiation Kit were obtained from Invitrogen (Carlsbad, CA, USA). Glutaraldehyde and paraformaldehyde (PFA) were obtained from Panreac (Barcelona, Spain).

### 2.2. Iron Oxide Magnetic Nanoparticles Synthesis

Iron oxide nanoparticles were synthesized using the co-precipitation of ferric and ferrous ions in an alkaline solution [62]. FeCl_2_·4H_2_O and FeCl_3_ at molar ratio 1:2 (Fe^2+^/Fe^3+^) were dissolved in 1.2 mL of ultrapure water. The obtained solution was heated at 90 °C under vigorous stirring (500 rpm). Then, 10 mL of 0.1 M NaOH solution was added dropwise to the solution under stirring with heating at 90 °C, resulting in dark-brown precipitate appearing during this step. The solution was stirred continuously for 30 min at 25 °C. The obtained precipitate was isolated by placing a strong magnet near the flask and washed five times with a citric acid solution (20 mg/mL). After that, nanoparticles were rinsed three times with distilled water. The resulting concentration of nanoparticles was measured by the gravimetric method (15.6 mg/mL). Additionally, the nanoparticles were functionalized with calf thymus DNA solution (50 µg/mL) in the presence of 0.1 M MgCl_2_ (Sigma Aldrich, Darmstadt, Germany), thus obtaining MNPs@DNA particles. DNA stock solution (2 mg/mL) in distilled water was prepared and subjected to ultrasound fragmentation with Bandelin SonoPlus sonifier (Bandelin, Berlin, Germany) as earlier described [63]. Briefly, DNA functionalization of particles was performed by mixing DNA solution with citrate-stabilized MNPs (3 mg/mL and 6 mg/mL) in the presence of MgCl_2_. The mixture was vigorously shaken for 1 min at room temperature. The resulting particles were further referred to as MNPs@DNA.

### 2.3. Nanoparticle Characterization

Hydrodynamic size and ζ-potential of particles were measured by dynamic light scattering (DLS) and electrophoretic light scattering methods (ELS) using a Zetasizer Nano ZS instrument (Malvern Panalytical Ltd., Malvern, UK). Size and ζ-potential measurements were conducted in disposable cuvettes (DTS1070) with a 1% water suspension of nanoparticles. All measurements were made in triplicate with an equilibration step of 60 s. The software was set to automatic acquisition mode. The morphology of MNPs was studied using atomic force microscopy (AFM). AFM images were obtained by scanning probe microscope Dimension Icon (Bruker, Billerica, MA, USA). The AFM samples were made as follows: the colloidal suspension of particles was diluted with ultrapure water, and 5 mL of the solution was deposited onto a clean glass slide with subsequent drying at room temperature. Operating was performed in PeakForce Tapping mode using ScanAsyst-Air cantilever (Bruker, Billerica, MA, USA; nominal length 115 mm, tip radius 2 nm and spring constant 0.4 N·m^−1^). The optimal parameters of scanning were set to obtain high-quality images of topography and nanomechanical characteristics. The peak force was set at 1–2 nN with a nominal line scan rate at 0.8–0.9 Hz and resolution 512–1024 lines per scan. The raw AFM data were processed using Nanoscope Analysis v.1.7. software (Bruker, Billerica, MA, USA). Hyperspectral dark-field analysis of particles was performed using an imaging system consisting of an Olympus BX51 upright microscope (Olympus, Tokyo, Japan) with a motorized stage module ProScan III (JH Technologies, Fremont, CA, USA), a CytoViva^®^ enhanced dark-field condenser (CytoViva, Auburn, Al, USA), a halogen light source (Fiber-Lite DC-950, 150 W; Dolan Jenner Industries Inc., Boxborough, MA, USA), an ImSpector V10E spectrograph (Specim, Oulu, Finland), and a Pco.Pixelfly usb CCD camera (PCO AG, Kelheim, Germany). Samples of MNPs were prepared by directly applying a drop of particle suspension to the clean microscope slide, and a coverslip lowered onto it. Dark-field images of MNPs were obtained using Exponent 7 image acquisition software (Dage-MTI, Michigan City, IN, USA). Hyperspectral data were captured in the wavelength range of 400 to 1000 nm with 2 nm spectral resolution at 0.25 s exposure and full illumination intensity using ENVI 4.8. software (Harris Geospatial Solutions, Broomfield, CO, USA). 

### 2.4. Glass Surface Coating

Modified glass substrates were obtained by self-assembly of magnetic nanoparticles on the glass surface. The 12 mm round borosilicate glass coverslips were treated in 2 M NaOH for 2 h and washed three times with distilled water. Then, the coverslips were placed in 0.1 M HCl solution, followed by three times rinsing with distilled water and drying at room temperature. After rinsing, the coverslips were sterilized with 70% ethanol and exposed to the UV light for 30 min. The immobilization of magnetic nanoparticles on glass surface was conducted by the drop deposition of 100 µL of particle solution (with concentrations of 3 mg/mL and 6 mg/mL) on the surface with subsequent drying for 24 h at room temperature. After drying, the samples were exposed to UV light for 30 min and washed three times with ultrapure water. As a result, four samples coated with either MNPs or MNPs@DNA at the concentration 3 or 6 mg/mL were obtained. 

### 2.5. Cells Cultivation

The mesenchymal stem cell culture obtained from adipose tissue was used. hTERT-transduced cells were obtained using lentiviral transduction. The pWPT-GFP construct (cat #12255, Addgene, Watertown, MA, USA) was applied to obtain hMSCs carrying the green fluorescent protein (GFP) gene. Then, cells with a high level of GFP expression were isolated using a FACS Aria III flow cytometer. Of 96 clones selected for each cell line, 8 monoclonal cell lines MSC-GFP were obtained, one of which was used in further experiments. The cells were cultured in Dulbecco’s modified eagle medium (DMEM) supplemented with 10% inactivated fetal bovine serum, antibiotics penicillin-streptomycin and 1% L-glutamine. The cells were cultured at 37 °C in an incubator with a relative air humidity of 80% and a CO_2_ level of 5%. Passaging of hTERT-transduced MSCs was carried out when the confluent growth reached 90% coverage. The cells were removed from culture vessels by keeping the cells for 7 min in 0.25% trypsin-EDTA solution with preliminary washing of cells with phosphate-buffered saline (PBS). Tali Image-Based Cytometer (Invitrogen, Waltham, MA, USA) was used for cell counting. 

### 2.6. Cells Analysis

Analysis of morphology and the number of adhered cells was determined by means of fluorescence microscopy. For this, hTERT-transduced MSCs at a density of 10^5^ cells per well were seeded onto a 6-well plate containing three samples of substrates each. After 16 h of cultivation under standard conditions, cell nuclei were stained with 4′,6-diamidino-2-phenylindole (DAPI; 300 nM), and actin filaments were stained with phalloidin (Phalloidin–tetramethylrhodamine B isothiocyanate; 50 µg/mL) accordingly to the manufacturers’ protocols (Sigma Aldrich, Germany). Cell counting and morphology analysis were performed using a Carl Zeiss Axio Imager Z2 fluorescence microscope (Carl Zeiss, Germany). 

### 2.7. Cell Viability

The MTT assay was performed to measure the time-dependent viability of cells cultured on modified surfaces according to the manufacturer’s protocol (Sigma Aldrich). Briefly, cells at a density of 2 × 10^3^ cells per well were plated onto 96-well plates, the wells of which had been pre-treated as described earlier for coverslips. The control wells remained untreated. The amounts of MNPs@DNA solutions (3 mg/mL and 6 mg/mL) were calculated based on the surface area of the well (0.32 cm^2^). After 24, 96 and 168 h of incubation, hMSCs were washed with phosphate-buffered saline (PBS) followed by the addition of 200 μL of fresh medium with 20 μL of MTT reagent. After 4 h of incubation at 37 °C, the medium was replaced with 200 μL of DMSO. The optical density values were measured at 540 nm using Multiskan™ FC Microplate Photometer (Thermo Fisher Scientific, Waltham, MA, USA).

### 2.8. Differentiation of hTERT-Transduced MSCs

To determine the ability of hMSCs to differentiate into adipocytes, chondrocytes and osteoblasts on modified glass, the cells in the amount of 10^5^ cells per well were cultured under standard conditions in 6-well plates. After reaching 90% of growth density, the culture medium was replaced with the appropriate differentiation medium.

For adipogenesis, hTERT-transduced MSCs were cultured in a DMEM medium containing 10% PBS with 0.5 mM isobutyl-methylxanthine, 1 µM dexamethasone, 200 µM indomethacin, and 10 µM recombinant insulin. The culture medium was replaced 2 times a week. Cell differentiation was studied using an optical microscope Carl Zeiss Primo Vert (Carl Zeiss, Germany). On the 14th and 21st days of cultivation, the lipid droplets of the cells were stained with Oil Red O. The cells were washed three times with phosphate buffer and fixed with 4% paraformaldehyde for 30 min. Further, the cells were incubated in 60% isopropanol solution for 10 s. Then, the Oil Red O (0.5% solution in isopropanol) with the addition of deionized H_2_O at a ratio of 6:4 was added to the cells for 15 min. After staining, cells were washed 5 times with deionized H_2_O.

For the induction of osteogenesis, cells were cultured in DMEM supplemented with 10% PBS, 0.1 µM dexamethasone, and 50 µM 2-phospho-L-ascorbic acid trisodium salt. The medium was replaced 2 times a week. On the 10th day of cultivation, 2 mM β-glycerophosphate disodium salt hydrate was added to the differentiation medium. On the 14th and 21st days of cultivation, the degree of osteogenesis was assessed by Von Koss staining. For this, the cells were fixed in 4% paraformaldehyde for 30 min and thoroughly washed with deionized H_2_O for 3 times. Then the cells were kept in a 2% silver nitrate solution for 10 min in a dark place, treated under ultraviolet light for 30 min, followed by rinsing thoroughly with deionized H_2_O.

Chondrogenesis was performed using the StemPro™ Chondrogenesis Differentiation Kit (Invitrogen, USA) according to the protocol provided by the manufacturer. The culture medium was replaced 2 times a week. Additionally, differentiation was carried out with a two-fold dilution of the chondrogenic medium with the DMEM nutrient medium. On the 14th and 21st days of cultivation, the cells were washed three times with PBS and fixed in a solution of 2.5% glutaraldehyde for 1 h. Then they were washed twice with PBS, and the proteoglycan of the cartilage tissue was stained with a 0.1% aqueous solution of safranin O for 15 min. After staining, cells were washed 5 times with deionized H_2_O.

The morphology and primary cell differentiation markers were examined using an optical microscope Carl Zeiss Primo Vert (Carl Zeiss, Germany). 

### 2.9. Statistics

The diameter of citrate-stabilized magnetic nanoparticles was manually calculated from 100 particles of raw AFM scans and represented as arithmetical mean ± standard deviation (SD). The root mean square of surface roughness in nanometers and coefficient of skewness were recorded from ≥3 randomly selected areas (20 × 20 µm) of each sample using Nanoscope Analysis v.1.7. software. Statistical analysis was conducted using GraphPad Prism v 8.4.3 (GraphPad Software, Inc., USA). The data were represented as arithmetical mean ± SD. The difference of parameters between unmodified surface and experimental samples was measured using Kruskal–Wallis one-way analysis of variance with Dunn’s post hoc test with statistical significance at *p* < 0.05. The number of cells on modified surfaces was calculated manually from 20 fluorescent microscopy images of DAPI and phalloidin-stained cells in culture at objective magnification ×10 and represented as arithmetical mean + SD.

## 3. Results

### 3.1. Characterization of Magnetic Iron Oxide Nanoparticles

**Figure 1:**
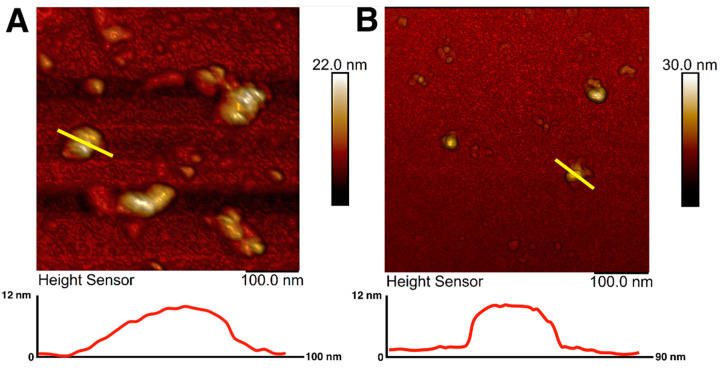
AFM images and surface profiles of magnetic nanoparticles without citrate ions stabilization (**A**) and with citrate ion stabilization (**B**). The yellow line shows the surface profile.

Magnetic iron oxide nanoparticles were synthesized using the chemical co-precipitation method and characterized for their colloidal variability and possible effects on resulting nanotopography. The prepared MNPs were coated with citrate ions to stabilize the particles in the dispersion [64]. The choice of citrate ions for MNPs coating was justified by their minimal effect on the size of the obtained nanoparticles and prevention of nanoparticle aggregation [65]. The sizes and morphology of magnetic nanoparticles were investigated using atomic force microscopy. AFM imaging allowed visualization of an aggregate of unstabilized MNPs as well as citrate-stabilized magnetic nanoparticles with irregular spherical morphology (Figure 1A,B). AFM scans showed that the diameter of the citrate-stabilized particles varied from 14 to 40 nm, which is in good agreement with the sizes of nanoparticles most used for surface modification [49]. The sizes of nanoparticles were additionally assessed using transmission electron microscopy (Supplementary Figure S1), which showed the average size of MNPs to be about 10 nm.

**Figure 2:**
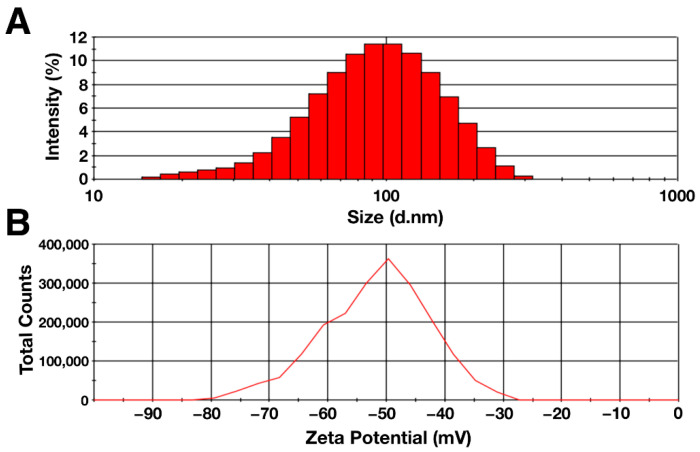
(**A**) Hydrodynamic diameter and (**B**) zeta potential of citrate-stabilized magnetic nanoparticles (0.1%).

The hydrodynamic diameter and zeta potential of the particles were determined using the dynamic light scattering method. The data obtained are shown in Figure 2. According to the data obtained, the hydrodynamic diameter of the synthesized citrate-stabilized magnetic nanoparticles had a distribution with negative symmetry. The average particle diameter was 80.5 ± 0.9, with the peak located in the range of 90–100 nm (Figure 2A). The zeta potential of the MNP aqueous solution was −50.4 ± 1.3 mV, indicating high colloidal stability of the solution (Figure 2B) [66]. The polydispersity index was 0.20 ± 0.01, which indicates the homogeneity of the suspension under study [67]. The differences in the sizes obtained with AFM and DLS were due to the difference in the counting method and the conditions in which the measurements take place. One of the factors is the presence of a hydration shell in the case of the DLS method, which can strongly influence the result [68]. In addition, the shape of the particles and the degree of homogeneity of the solution may affect the hydrodynamic diameter [69,70].

### 3.2. Characteristics of Modified Substrates

**Figure 3:**
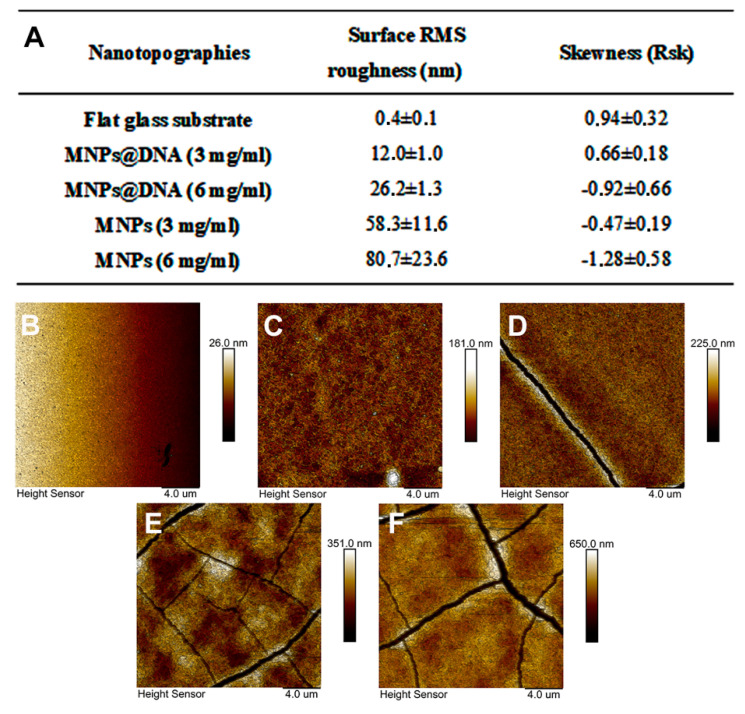
(**A**) Surface RMS roughness and their asymmetry coefficient obtained with AFM. AFM surface topography: (**B**) glass, (**C**) MNPs@DNA 3 mg/mL, (**D**) MNPs@DNA 6 mg/mL, (**E**) MNPs 3 mg/mL, (**F**) MNPs 6 mg/mL.

To modify the surface of the glass, a simple method of nanomaterial immobilization was used, which was based on placing a drop of nanoparticles solution followed by subsequent drying (drop-casting method). A solution of negatively charged citrate-stabilized magnetic nanoparticles was pre-mixed with DNA in the presence of magnesium ions. DNA molecules can bind magnetic nanoparticles without preliminary modification [71]. In addition, it was shown that the immobilization of DNA molecules on an unmodified glass surface is possible using UV radiation with a wavelength of 254 nm [72]. The addition of magnesium ions made it possible to form cation bridges [73] or neutralize the charges of the phosphate group of DNA and the carboxyl group of the MNPs, thereby reducing the energy barrier between the particles and DNA [74]. The topography of the modified surfaces was investigated using AFM (Figure 3B–F). The images show the topography of unmodified glass and surfaces coated with magnetic nanoparticles with and without DNA. The nanofilm structure is the result of magnetic nanoparticles aggregation into clusters with complex morphology. The MNPs@DNA (3 and 6 mg/mL) samples differed in maximum height by ≈40 nm. The height of the samples without the addition of DNA was 2 and 4 times higher relative to the surface of MNPs@DNA 3 mg/mL. Cracking could also be seen on all samples except MNPs@DNA 3 mg/mL. 

Surface roughness was characterized using the RMS roughness (Sq) and asymmetry (Ssk) parameters. The obtained Sq values confirmed the previous observations: the roughness increased with increasing particle concentration (Figure 3A). In addition, the roughness index was also influenced by DNA, in the absence of which Sq increased several times.

**Figure 4:**
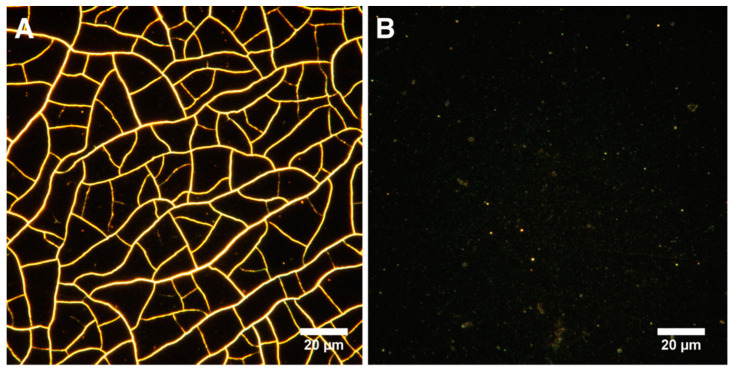
Visualization of surfaces with dark-field microscopy: (**A**) MNPs 3 mg/mL and (**B**) MNPs@DNA 3 mg/mL.

Samples with DNA in their composition had significantly less rough surfaces. The asymmetry parameter characterizes the surface morphology. A negative value indicates a predominance of dents and cracks. Conversely, a positive value indicates a predominance of peaks and protrusions on the surface [75]. According to the results of AFM data analysis, Ssk was negative on all surfaces, except for the control sample and MNPs@DNA 3 mg/mL. Moreover, the highest absolute value was observed for MNPs and MNPs@DNA samples with the concentration of 6 mg/mL, which indicates a possible low stability of the coating. It was also confirmed by surface cracks observed on AFM images (Figure 3). Atomic force microscopy showed the formation of efficient nanotopography in MNPs@DNA 3 mg/mL samples. Additionally, dark-field microscopy examined the structure of surfaces with a larger area, where the uniform distribution of particles in the MNPs@DNA 3 mg/mL sample was noticeable (Figure 4).

### 3.3. Cell Morphology

**Figure 5:**
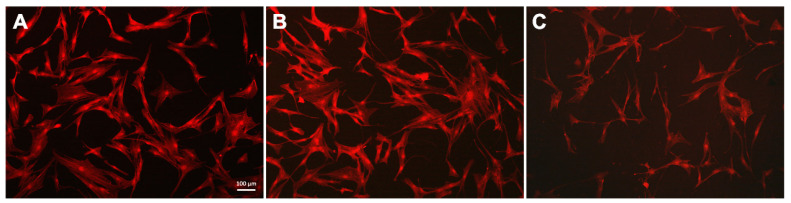
Fluorescent images of hTERT-transduced MSCs with stained cytoskeleton: (**A**) glass, (**B**) MNPs@DNA 3 mg/mL and (**C**) MNPs@DNA 6 mg/mL. F-actin stained with phalloidin (red).

hTERT-transduced mesenchymal stem cells were cultured on the obtained substrates with DNA and magnetic nanoparticles coatings for 24 h. Staining the f-actin in the cytoplasm made it possible to assess the cell morphology (Figure 5) and was also used as an indicator of the hTERT-transduced MSCs state [76]. The studied cells had a typical fusiform shape: on the untreated glass surface and the MNPs@DNA 3 mg/mL coated sample, f-actin bundles formed contractile stress fibers. On the MNPs@DNA 6 mg/mL coated sample, which had chaotic surface cracks, the total number of cells was lower, indicating a decrease in adhesion. The direction of cell growth did not depend on the direction of microcracks on the surface coated with MNPs@DNA 6 mg/mL.

The total number of cells on smooth and modified surfaces was counted. The average number of cells on the smooth glass was 113 ± 3.2, while on the surfaces modified with MNPs@DNA it was 3 and 6 mg/mL—114.7 ± 8.7 and 75.3 ± 9.5, respectively. The cells can investigate the stiffness of the substrate, transmitting mechanical signals from the substrate through adhesion receptors that activate downstream signals regulating both the extent of cell proliferation and growth rate [77]. In addition, the viability of cells cultured on different substrates for 96 h was assessed. No significant differences in cell viability were observed between variants (Supplementary Figure S2).

### 3.4. Cell Differentiation

The chondrogenic, adipogenic and osteogenic differentiation of hMSCs cultivated on surfaces modified with MNPs in different concentrations was investigated. Qualitative staining showed that, both on smooth surfaces and on surfaces with complex nanotopography formed by magnetic nanoparticles and DNA, the cells differentiated into all studied phenotypes.

**Figure 6:**
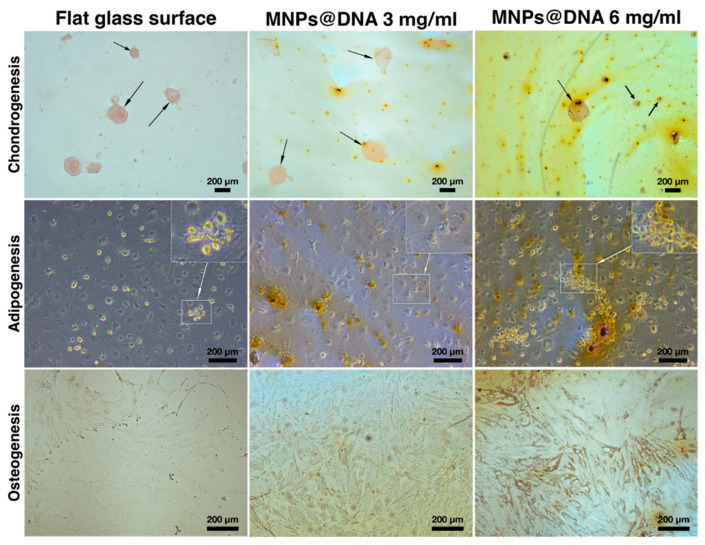
Differentiation of hTERT-transduced MSCs on 14 days of cultivation: chondrocytes were stained with safranin O, adipocytes—with Oil Red O, and osteoblasts—with silver nitrate (von Kossa stain). Arrows indicate chondrocyte aggregates. Areas of cells with lipid droplets are marked with squares.

The differentiation of cells into chondrocytes was visually determined by staining the proteoglycans of the extracellular matrix with safranin O. The staining showed that chondrogenic differentiation and micro-aggregation of cells incubated in chondrogenic medium successfully occurred on all surfaces. To assess adipogenesis, the cells were stained with Oil Red O on day 14 after cell transfer to differentiation medium. Small agglomerates of lipid droplets were found in the cells sampled from all surfaces. The formation of tiny lipid droplets indicated that adipocytes did not reach their final maturity on the 14th day of differentiation (Figure 6, middle row). In the case of differentiation of stem cells into osteoblasts, an increase in the synthesis of calcium deposits was observed as the MNPs contents in the cell support increased (Figure 6, bottom row). Compared to the unmodified surface, the mineralization of cells on the DNA@MNPs treated surfaces was more evident and distinguishable, indicating osteogenesis induction.

To study the potential of nanostructured surfaces to induce cell differentiation at lowered level of differentiation promoting growth factors, the concentration of growth factors was decreased two-fold by the growth medium dilution, and MSCs differentiation on nanostructured surfaces was assessed. 

**Figure 7:**
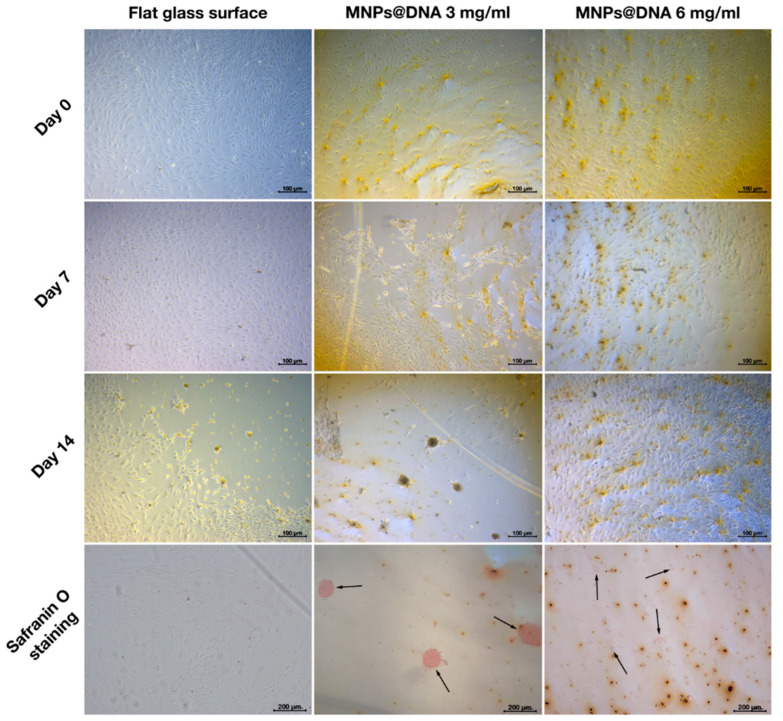
Chondrogenic differentiation of hMSCs on surfaces coated with a solution of 3 and 6 mg/mL of magnetic nanoparticles with DNA (50 µg/mL). The chondrocytes were stained with safranin O. The arrows show the aggregates of chondrocytes.

In the case of adipogenesis and osteogenesis, there were no visible changes in the differentiation process (data not shown). However, differences were observed between control and nanostructured surfaces in the induction of chondrogenic MSCs differentiation in diluted growth medium. On day 7 of cell incubation in diluted growth medium, an early condensation of cells was noticeable on the coating prepared from MNPs@DNA 3 mg/mL solution, in contrast to the control (uncoated glass) substrate (Figure 7). Safranin staining showed successful differentiation of cells into chondrocytes on substrates coated with magnetic nanoparticles with DNA, being more intense on the surfaces deposited from MNPs@DNA 3 mg/mL solution. The cells of the control group were slightly stained. It was clear that cellular aggregates were formed on the 14th day of differentiation. Thus, MNPs@DNA supports were capable of successful induction of chondrogenic stem cell differentiation even at a decreased level of growth factors. 

## 4. Discussion

MSCs have great potential as a therapeutic instrument for the restoration of the musculoskeletal system, since they can differentiate into myocytes, chondrocytes, adipocytes and osteoblasts and thus can be used for the construction of cartilage, bone, adipose and muscle tissues [78,79]. The ability of MSCs to differentiate in response to external mechanical stimuli implies that MSCs differentiation can potentially be promoted in vitro by application of artificial nanostructured substrates [23]. It was shown that nanotopographic cues from underlying materials exert potent effects on the osteogenic differentiation of MSCs because of their niche-mimicking features [80] and the osteogenic differentiation of stem cells can be accelerated by substrates with anisotropic structures [81]. The aim of our work was to study the ability of a nanorough surface coating formed from magnetic nanoparticles and DNA to stimulate the differentiation of hTERT-transduced MSCs.

Magnetic nanoparticles are actively used in cell biology as labels for tracking cells in vivo using magnetic resonance imaging [82]. Due to their paramagnetic properties they can also be used as containers for targeted drug delivery [83,84], cancer therapy [85,86], biovisualization and cell separation [87]. Recently, it has also been demonstrated that magnetic nanoparticles have great potential in the field of tissue engineering. Magnetic-field-driven assembly of MNPs into complex patterns was used for producing nanostructured tissue scaffolds capable of stimulating the differentiation of cells into osteoblasts [11]. The doping of nanofibers or scaffolds with magnetic nanoparticles also increased the growth and differentiation of MSCs [50].

In our previous work, we developed surfaces coated with three nanomaterials of different morphology and composition: magnetic iron oxide nanoparticles, halloysite nanotubes, and graphene oxide [88]. Among these, the magnetic nanoparticles have proved to be the most attractive nanomaterial due to their small size and the uniform topography of the surface formed. However, the deposition of magnetic nanoparticles on glass without any additional modifications led to the formation of a coating that was not stable and thus not suitable for differentiation. Therefore, DNA was added to the nanoparticles in the presence of magnesium ions. The effect of MNPs@DNA-modified surfaces on the differentiation of hTERT-transduced MSCs into chondrocytes, adipocytes, and osteoblasts was studied. A successful differentiation of the cells into all three cell types showed that the hTERT-transduced adipose-derived MSCs met the morphofunctional criteria.

The MNPs@DNA surfaces produced were capable of inducing osteogenic and chondrogenic cell differentiation (Figure 6). The inclusion of DNA as an embedding matrix was beneficial for the nanotopography, stability and the cell differentiating potential of the obtained substrates. A successful osteogenic differentiation of MSCs on the MNPs@DNA surfaces was evidenced by a higher calcium deposition. This method is widely used to indicate osteogenic cell differentiation [89,90], along with such methods as assessment of alkaline phosphatase activity [91,92] and expression of osteogenesis-related genes [93,94]. The ability of DNA to promote osteogenesis was previously demonstrated during its use as a component of coatings for dental and bone implants [95,96,97,98]. The presence of magnesium could also contribute to cell differentiation due to its role in the regulation of osteogenesis [99]. The osteogenic potential of DNA-based coatings was explained by the release of phosphate ions during DNA degradation and upregulation of the expression of sodium-dependent phosphate co-transporter by phosphate [100]. 

In addition to DNA, MNPs present in composite coatings could exert their own intrinsic cell differentiation promoting properties. MNPs-based composite scaffolds can enhance cell adhesion and osteogenesis, both by increasing the surface area of the substrate and by penetration of individual particles into cells [49]. The assumed mechanisms include the activation of ion channels by internalized iron oxide particles and mechanical stress caused by the interaction of MNPs with the cell membrane [101,102]. 

However, not only the surface composition but its nanotopography and mechanical properties also influenced the cell adhesion and differentiation. As demonstrated by atomic force and dark-field microscopy, the MNPs@DNA sample of 3 mg/mL had a uniform particle distribution over the entire surface, while the surface with higher MNPs contents was rougher and had some cracks on it. Significantly reduced cell adhesion at a high content of magnetic nanoparticles in the MNPs@DNA composite was probably related to the high surface roughness. This is consistent with earlier studies where reduced attachment of cells to rougher surfaces was demonstrated [103,104]. Poor surface attachment resulted in lower cell counts on the surface coated with DNA, containing a high concentration of MNPs. 

A glass surface modified with magnetic nanoparticles (3 mg/mL) and DNA induced active chondrogenesis of hTERT-transduced MSC at a reduced level of growth factors, unlike a smooth surface or a surface with higher nano-roughness (Figure 7). The ability of MSCs to differentiate into chondrocytes has been demonstrated previously on titanium-coated substrates with a similar surface roughness index [105]. Wu et al. [106] found that, compared to non-patterned surfaces, nanotopography of the surface in the form of nanopillars and nanoholes caused changes in the morphology and structure of the cytoskeleton and induced chondrogenesis of MSCs. Additionally, chondrogenesis began earlier on modified surfaces than on clear glass for the cultivation of cells in a twice-diluted differentiation medium. 

The development of approaches allowing the reduced use of growth factors in tissue engineering is important, because growth factors can be degraded in vivo by proteolytic enzymes necessitating their constant replenishment or initial application of excessive doses. Moreover, ample use of growth factors increases production costs and can result in side-effects, such as malignant cell transformations [107]. As a result of our work, it was shown that combining magnetic nanoparticles and DNA in a coating provides favorable conditions for cell chondrogenesis and osteogenesis and can partly substitute the action of chondrogenesis-inducing growth factors. Thus, the combination of MNPs and DNA can be useful for making cell-differentiation-promoting substrates for laboratory studies or therapeutic purposes. Moreover, various synthesis methods can be used to produce magnetic particles of different sizes and shapes [108] while the superparamagnetic properties of MNPs allow their manipulation with an external magnetic field [42] and generate substrates with controlled unique surface nanotopographies [109]. 

## 5. Conclusions

We obtained glass substrates modified with magnetic nanoparticles. The addition of DNA to the composition of the applied solution made it possible to increase the coating stability. Surface nanotopography, which was generated with a 3 mg/mL solution of magnetic nanoparticles with the addition of DNA, promoted the differentiation of cells into chondrocytes under conditions of reduced growth factor concentration. We observed a similar beneficial effect of the coatings on cell differentiation into osteoblasts, resulting in increased matrix mineralization. The positive effect of the magnetic nanoparticle coating on the differentiation of hTERT-transduced MSCs makes it possible to regard this nanomaterial as a prospective basis for the creation and study of structures with unique surface nanotopography for manipulating the growth of cells.

## Data Availability

Not applicable.

## Supplementary Materials

The following supporting information can be downloaded at: https://www.mdpi.com/article/10.3390/polym14020344/s1, Figure S1: Citrate-stabilised MNPs visualised with transmission electron microscopy. Figure S2: Viability of hTERT-transduced mesenchymal stem cells grown on surfaces modified with DNA and magnetic nanoparticles.
