# Supplementary material for: DNA/Magnetic Nanoparticles Composite to Attenuate Glass Surface Nanotopography for Enhanced Mesenchymal Stem Cell Differentiation
Source: Polymers (Basel). 2022 Jan 17;14(2):344. doi: 10.3390/polym14020344 (PMC8779295; doi:10.3390/polym14020344)
Supplement: Supplementary file 1 [file polymers-14-00344-s001.zip › polymers-1477144-supplementary.pdf]

polymers MDPI

Supplemental Materials

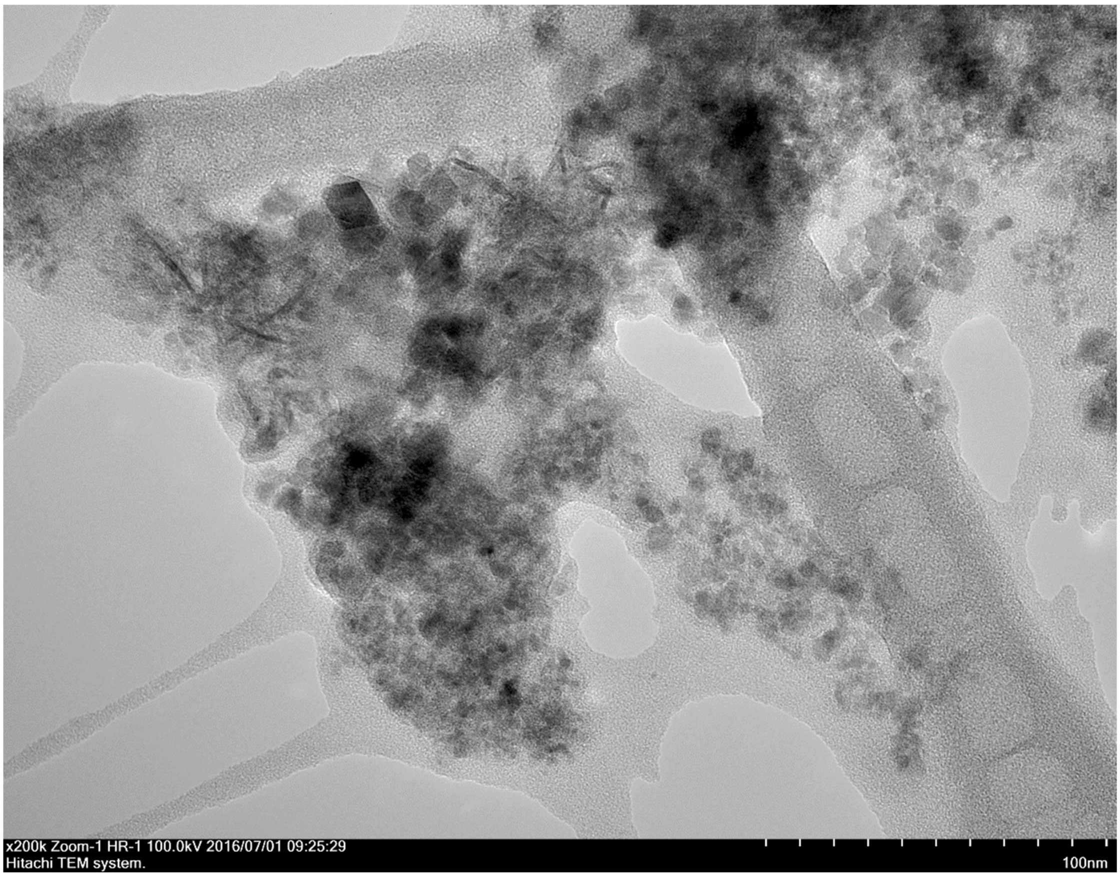

Figure S1. Citrate-stabilised MNPs visualised with transmission electron microscopy.

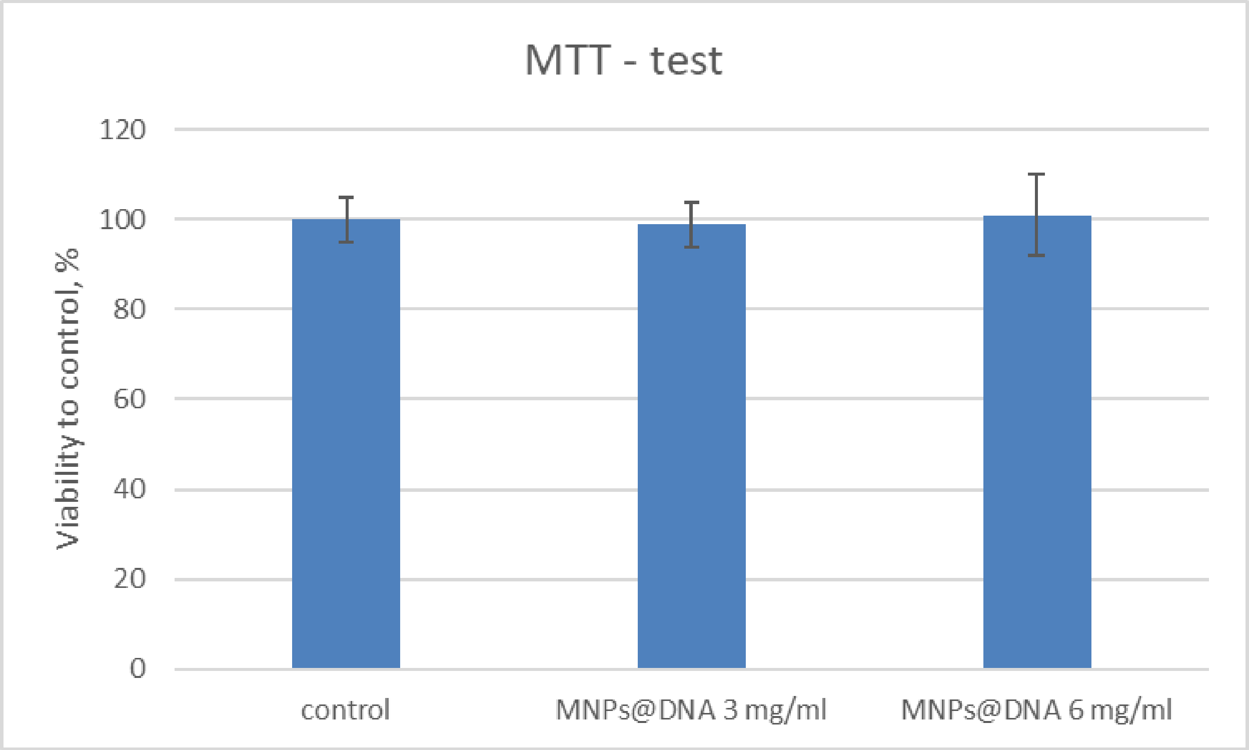

Figure S2. Viability of hTERT-transduced mesenchymal stem cells grown on surfaces modified with DNA and magnetic nanoparticles.
